# Polyenylpyrrole Derivatives Inhibit NLRP3 Inflammasome Activation and Inflammatory Mediator Expression by Reducing Reactive Oxygen Species Production and Mitogen-Activated Protein Kinase Activation

**DOI:** 10.1371/journal.pone.0076754

**Published:** 2013-10-07

**Authors:** Kuo-Feng Hua, Ju-Ching Chou, Yulin Lam, Yu-Ling Tasi, Ann Chen, Shuk-Man Ka, Zhanxiong Fang, May-Lan Liu, Feng-Ling Yang, Yu-Liang Yang, Yi-Chich Chiu, Shih-Hsiung Wu

**Affiliations:** 1 Department of Biotechnology and Animal Science, National Ilan University, Ilan, Taiwan; 2 Department of Chemistry, National University of Singapore, Singapore, Singapore; 3 Graduate Institute of Life Science, National Defense Medical Center, Taipei, Taiwan; 4 Department of Pathology, Tri-Service General Hospital, National Defense Medical Center, Taipei, Taiwan; 5 Graduate Institute of Aerospace and Undersea Medicine, National Defense Medical Center, Taipei, Taiwan; 6 Department of Nutritional Science, Toko University, Chiayi, Taiwan; 7 Institute of Biological Chemistry, Academia Sinica, Taipei, Taiwan; 8 Agricultural Biotechnology Research Center, Academia Sinica, Taipei, Taiwan; 9 Department of Biomechatronic Engineering, National Ilan University, Ilan, Taiwan; Vanderbilt University School of Medicine, United States of America

## Abstract

Two polyenylpyrroles from a soil ascomycete *Gymnoascus reessii* were previously identified as hit compounds in screening for cytotoxicity against lung cancer cells. These compounds and various analogs, which have been previously synthesized and tested for anti-lung cancer cell activity, were tested for anti-inflammatory activity. After preliminary screening for cytotoxicity for RAW 264.7 murine macrophage cells, the non-toxic compounds were tested for anti-inflammatory activity using lipopolysaccharide (LPS)-activated RAW 264.7 cells. Compounds **1h**, **1i**, and **1n** reduced LPS-induced nitric oxide (NO) production, with respective ED_50_ values of 15 ± 2, 16 ± 2, and 17 ± 2 µM. They also reduced expression of inducible NO synthase and interleukin-6 (IL-6) without affecting cyclooxygenase-2 expression. Compound **1h** also reduced secretion of IL-6 and tumor necrosis factor-α by LPS-activated J774A.1 murine macrophage cells, primary mice peritoneal macrophages, and JAWSII murine bone marrow-derived dendritic cells and reduced NLRP3 inflammasome-mediated interleukin-1β (IL-1β) secretion by LPS + adenosine triphosphate-activated J774A.1 and JAWSII cells. The underlying mechanisms for the anti-inflammatory activity of compound **1h** were found to be a decrease in LPS-induced reactive oxygen species (ROS) production, mitogen-activated protein kinase phosphorylation, and NF-κB activation and a decrease in ATP-induced ROS production and PKC-α phosphorylation. These results provide promising insights into the anti-inflammatory activity of these conjugated polyenes and a molecular rationale for future therapeutic intervention in inflammation-related diseases. They also show how compound **1h** regulates inflammation and suggest it may be a new source for the development of anti-inflammatory agents to ameliorate inflammation- and NLRP3 inflammasome-related diseases.

## Introduction

One of the geological characteristics of Taiwan is its abundant geothermal resources, which provide a special environment for the growth of certain microorganisms that have been utilized in various scientific and industrial areas [1]. Under normal growth conditions, fungi produce useful metabolites, such as penicillin and immunosuppressive compounds that have improved the quality of human life, and these organisms are still a rich source of bioactive compounds [2-4]. In addition, the extreme growth conditions of thermophilic fungi also allow them to synthesize novel metabolites.

Conjugated polyenes are an interesting class of widely occurring natural polyketides with useful biological properties, such as antibacterial [5], antifungal [5,6], and antitumor [7] activities. Previously, auxarconjugatin A and *12E*-isorumbrin isolated from the soil ascomycete *Gymnoascus reessii* and demonstrated that *12E*-isorumbrin had antitumor activity [8]. We then synthesized auxarconjugatin A and *12E*-isorumbrin and various analogs and evaluated them for anti-tumor activity, and identified two compounds with high cytotoxicity (active at nM levels) for the human non-small cell lung carcinoma cell line A549 [9]. Since polyketides also exhibit potent immune modulating activities [10,11], making them potential resources for the discovery of new immune modulating drugs, in the present study, we evaluated these compounds for anti-inflammatory activity.

The innate immune response is typically triggered by pathogen-associated molecular patterns shared by groups of different microbial pathogens and recognized by toll-like receptors (TLRs) or other receptors expressed on the cell surface of immune cells [12]. Lipopolysaccharide (LPS), a pathogen-associated molecular pattern molecule produced by Gram-negative bacteria, can induce production of inflammatory mediators, such as nitric oxide (NO), tumor necrosis factor-α (TNF-α), interleukin (IL)-6, and IL-1β, by binding to TLR4 [13]. Unlike other cytokines, IL-1β is synthesized as an inactive immature form (precursor of IL-1β, proIL-1β) via transcriptional activation in activated macrophages [14]. ProIL-1β is cleaved into IL-1β by active caspase 1, generated by the NLRP3 inflammasome, a multi-protein complex [15,16]. The NLRP3 inflammasome controls disease progression and inflammatory responses, such as those caused by infection [17-19], obesity [20], cholesterol crystals [21],, silica crystals [22], amyloid-beta [23], and uric acid crystals [24]. Recent findings suggest that ROS regulate NLRP3 inflammasome activation and TLR4 signaling [14,25,26] and that inhibition of NLRP3 activation may be a therapeutic strategy for inflammation-related diseases [27].

In this study, we evaluated the anti-inflammatory activity of the synthesized polyenylpyrroles and analogs using LPS-activated macrophages and identified compound **1h** as a non-toxic compound that can reduce inflammatory mediator expression and NLRP3 inflammasome activation. The intracellular signaling pathways affected by compound **1h** in activated macrophages were also investigated.

## Materials and Methods

### Materials

**Figure 1:**
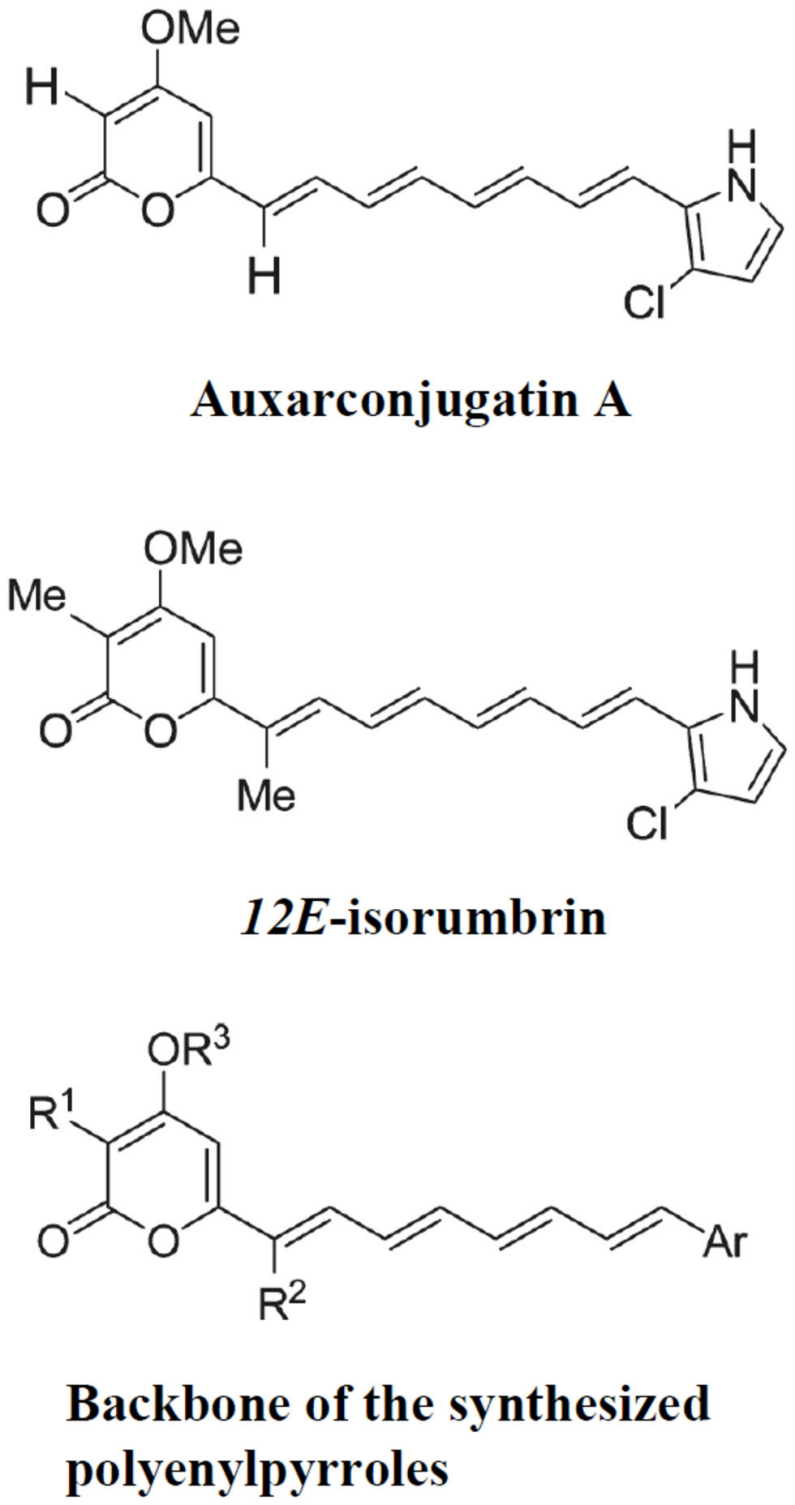
Backbone of the synthesized polyenylpyrroles.

The polyenylpyrroles, auxarconjugatin A and *12E*-isorumbrin, and a range of analogues (Figure 1 and Table 1) were synthesized as described previously [9]. The backbone of the synthesized polyenylpyrroles is shown in Figure 1. The compounds were dissolved in DMSO and used in cultures at a final concentration of 0.1% DMSO. LPS (from *Escherichia coli* 0111:B4), adenosine triphosphate (ATP), and mouse antibodies against mouse phospho-ERK1/2, phospho-JNK1/2, phospho-p38, or actin were purchased from Sigma (St. Louis, MO). Rabbit antibodies against mouse phospho-IKK-α/β, IKK, phospho-IκB-α, IκB-α, phospho-PKC-α, IL-1β, caspase-1, inducible NO synthase (iNOS), cyclooxygenase-2 (COX-2), or phospho-IKK-α/β and horseradish peroxidase (HRP)-labeled second antibodies were obtained from Santa Cruz Biotechnology (Santa Cruz, CA). Mouse monoclonal anti-mouse NLRP3 antibody was purchased from Enzo Life Sciences, Inc. (Farmingdale, NY). IL-1β, TNF-α, and IL-6 ELISA kits were purchased from R&D Systems (Minneapolis, MN).

**Table 1:** Cytotoxicity of, and inhibition of LPS-induced NO production by, polyenylpyrrole derivatives in RAW264.7 macrophages.

Sample	R^1^	R^2^	R^3^	Ar	IC_50_^a^	ED_50_
1a	H	H	Me	3-chloropyrrol-2-yl	< 10 µM	N.D.
1b	Me	H	Me	3-chloropyrrol-2-yl	< 10 µM	N.D.
1c	*n*Bu	H	Me	3-chloropyrrol-2-yl	< 10 µM	N.D.
1d	H	Me	Me	3-chloropyrrol-2-yl	< 10 µM	N.D.
1e	Me	Me	Me	3-chloropyrrol-2-yl	< 10 µM	N.D.
1f	Me	Et	Me	3-chloropyrrol-2-yl	< 10 µM	N.D.
1g	*n*Bu	Me	Me	3-chloropyrrol-2-yl	< 10 µM	N.D.
1h	H	H	H	3-chloropyrrol-2-yl	> 100 µM	15±2 µM
1i	Me	H	H	3-chloropyrrol-2-yl	> 100 µM	16±2 µM
1j	H	H	Me	3-chlorothiophen-2-yl	> 100 µM	29±6 µM
1k	Me	H	Me	3-chlorothiophen-2-yl	> 100 µM	18±2 µM
1l	Me	H	Me	2-chlorophenyl	> 100 µM	18±3 µM
1m	Me	H	Me	3-chlorophenyl	> 100 µM	26±3 µM
1n	Me	H	Me	3-chloro-1-mesyl-pyrrol-2-yl	> 100 µM	17±2 µM

a IC_50_ value expressed as the mean value for triplicate wells from at least three experiments using the AlamarBlue® assay.

N.D.: non-determined.

### Cell culture

The murine macrophage cell lines RAW 264.7 and J774A.1 and the C57BL/6 murine bone marrow-derived dendritic cell line JAWSII were purchased from the American Type Culture Collection. RAW 264.7 macrophages stably transfected with the NF-κB reporter gene (RAW-Blue™ cells) were purchased from InvivoGen (San Diego, CA). RAW 264.7, J774A.1, and RAW-Blue™ cells were grown in RPMI-1640 medium supplemented with 10% heat-inactivated fetal bovine serum (FBS) (both from Life Technologies, Carlsbad, CA), while JAWSII cells were grown in RPMI-1640 medium supplemented with 20% non-inactivated FBS and 5 ng/ml of murine GM-CSF (R&D Systems). All cells were cultured at 37 °C in a 5% CO_2_ incubator.

### AlamarBlue® assay for cell viability

RAW 264.7 cells were seeded at a density of 5000 cells in 100 µl of RPMI 1640 medium containing 10% heat-inactivated FBS in each well of 96-well flat-bottom plates and incubated for 24 h at 37 °C in a 5% CO_2_ incubator, then for 24 h with the test samples, then AlamarBlue® assay kits (AbD Serotec Ltd.) were used to measure cytotoxicity using the protocol described by the manufacturer.

### Enzyme-linked immunosorbent assay (ELISA)

The effects of the test samples on IL-1β, TNF-α, and IL-6 production were measured by ELISA according to the manufacturer’s instructions. In brief, 50 µl of biotinylated antibody reagent and 50 µl of supernatant were added to a stripwell plate precoated with anti-mouse IL-1β, TNF-α, or IL-6 antibodies, which was then incubated at room temperature for 2 h. After washing the plate three times with washing buffer, 100 µl of diluted streptavidin-HRP concentrate was added to each well and the plate incubated at room temperature for 30 min. The washing process was repeated, then 100 µl of a premixed tetramethylbenzidine substrate solution was added to each well and the plate incubated at room temperature in the dark for 30 min. After addition of 100 µl of stop solution to each well, the absorbance at 450 nm of each well was measured on a microplate reader.

### NO production inhibitory assay

RAW 264.7 cells seeded in 24-well plates at a density of 5 × 10^5^ cells/ml (1 ml) were incubated for 24 h with or without LPS (1 µg/ml) in the absence or presence of the test samples, then NO production was measured indirectly by analysis of nitrite levels using the Griess reaction.

### NF-κB reporter assay

RAW-Blue™ cells, RAW 264.7 macrophages stably expressing the gene for secreted embryonic alkaline phosphatase (SEAP) inducible by NF-κB, were seeded in 60 mm dishes at a density of 5 × 10^5^ cells/ml (1 ml) and grown overnight in a 5% CO_2_ incubator at 37 °C. They were then pretreated with vehicle or compound **1h** for 30 min, then LPS (1 µg/ml) was added and incubation continued for 24 h. The medium was then harvested and 20 µl aliquots mixed with 200 µl of QUANTI-Blue™ medium (InvivoGen) in 96-well plates and incubated at 37 °C for 15 min, then SEAP activity was assessed by measuring the optical density at 655 nm using an ELISA reader.

### Western blots

Cells were washed twice with ice-cold phosphate-buffered saline (PBS) and lysed on ice for 5 min with lysis buffer (20 mM Tris-HCl, pH 7.5, 150 mM NaCl, 1 mM Na _2_EDTA, 1 mM EGTA, 1% Triton X-100, 2.5 mM sodium pyrophosphate, 1 mM beta-glycerophosphate, 1 mM Na _3_VO_4_, 1 µg/ml of leupeptin, 1 mM PMSF, protease inhibitor cocktail). The lysate was then centrifuged at 12,000 x *g* at 4 °C for 10 min and the pellet discarded. Proteins in the supernatant were separated by SDS-PAGE and electrotransferred to a PVDF membrane (EMD Millipore Corporation), which was then blocked by incubation for 1 h at room temperature in blocking buffer [(5% nonfat milk in PBS containing 0.1% Tween 20 (PBST)], then incubated for 2 h at room temperature with the primary antibody diluted in blocking buffer. After three washes in PBST, the membrane was incubated for 1 h at room temperature with an appropriate HRP-conjugated secondary antibody diluted in blocking buffer and developed using an enhanced chemiluminescence Western blot detection system (EMD Millipore Corporation).

### Measurement of intracellular ROS production

Intracellular ROS production was measured by detecting the fluorescence intensity of the 2’, 7’-dichlorofluorescein, the oxidation product of 2’, 7’-dichlorofluorescein diacetate (Molecular Probes, Eugene, OR). In one study, RAW 264.7 macrophages (5 × 10^5^/ml; 0.1 ml) were incubated with vehicle, compound **1h** (20 µM), or NAC (10 mM) for 30 min, then 2’, 7’-dichlorofluorescein diacetate (2 µM) was added for 30 min, then LPS was added for the indicated time. In another, J774A.1 macrophages (5 × 10^5^/ml; 0.1 ml) were incubated with LPS (1 µg/ml) for 6 h, then vehicle, compound **1h** (20 µM), or DPI (25 µM) was added for 30 min. 2’, 7’-dichlorofluorescein diacetate (2 µM) was then added for 30 min before incubation with ATP (5 mM) for the indicated time; while, in another, the order of addition of compound1h/DPI and LPS was reversed. The fluorescence intensity of 2’, 7’-dichlorofluorescein was detected at an excitation wavelength of 485 nm and an emission wavelength of 530 nm on a microplate absorbance reader (Bio-Rad Laboratories, Inc).

### Measurement of NF-κB p65 nuclear translocation

Nuclear proteins were extracted from RAW 264.7 and J774A.1 cells using a Nuclear Extract Kit (Active Motif) according to the manufacturer’s instructions and nuclear NF-κB p65 activation quantified using an ELISA-based TransAM NF-κB kit (Active Motif, Tokyo, Japan) according to the manufacturer’s protocol by reading the absorbance at 450 nm with a microplate absorbance reader (Bio-Rad Laboratories, Inc) and a reference wavelength of 655 nm.

### Statistical analysis

All values are given as the mean ± SD. Data were analyzed by one-way ANOVA with a subsequent Scheffé test.

## Results

### Synthesis of the test compounds

The polyenylpyrroles, auxarconjugatin A and *12E*-isorumbrin, were previously isolated from the soil ascomycete *Gymnoascus reessii* and *12E*-isorumbrin shown to be cytotoxic for various cancer cells [8]. We have synthesized these compounds and a range of analogs (Table 1 and Figure 1) and investigated their cytotoxicity for the human lung cancer sell line A549 [9]. The backbone of the synthesized polyenylpyrroles is shown in Figure 1. Since the 3-chloropyrrole group has been shown to play an important role in the cytotoxicity of auxarconjugatin A (compound **1b**) and *12E*-isorumbrin (compound **1e**) [8], in some analogs, it was replaced by other 2- or 3-chlorosubstituted aromatic rings. In addition, H, Me, Et, or *n*-Bu was added at the different R positions (Table 1).

### Effect of polyenylpyrrole derivatives on macrophage viability

The aim of the present study was to identify non-toxic polyenylpyrrole derivatives that could be used as anti-inflammatory agents. Compounds 1a-n (Table 1) at concentrations from 6.25 µM to 100 µM were evaluated for cytotoxicity against the murine macrophage cell line RAW 264.7 after 24 h treatment. As shown in Table 1, compounds **1a-g** exhibited high cytotoxicity, with IC_50_ values below 10 µM, indicating that they were not suitable for evaluation of their anti-inflammatory activities. In contrast, compounds **1h-n** were not cytotoxic at any of the concentrations tested and their anti-inflammatory activity was therefore examined by measuring their ability to reducing LPS-induced NO production by RAW 264.7 macrophages. As shown in Table 1, the three most potent compounds were **1h, 1i**, and **1n**, with respective ED_50_ values of 15 ± 2, 16 ± 2, and 17 ± 2 µM, and these were used in subsequent studies.

### Compounds 1h, 1i, and 1n decrease production of NO, iNOS, and IL-6 by LPS-activated RAW 264.7 macrophages

**Figure 2:**
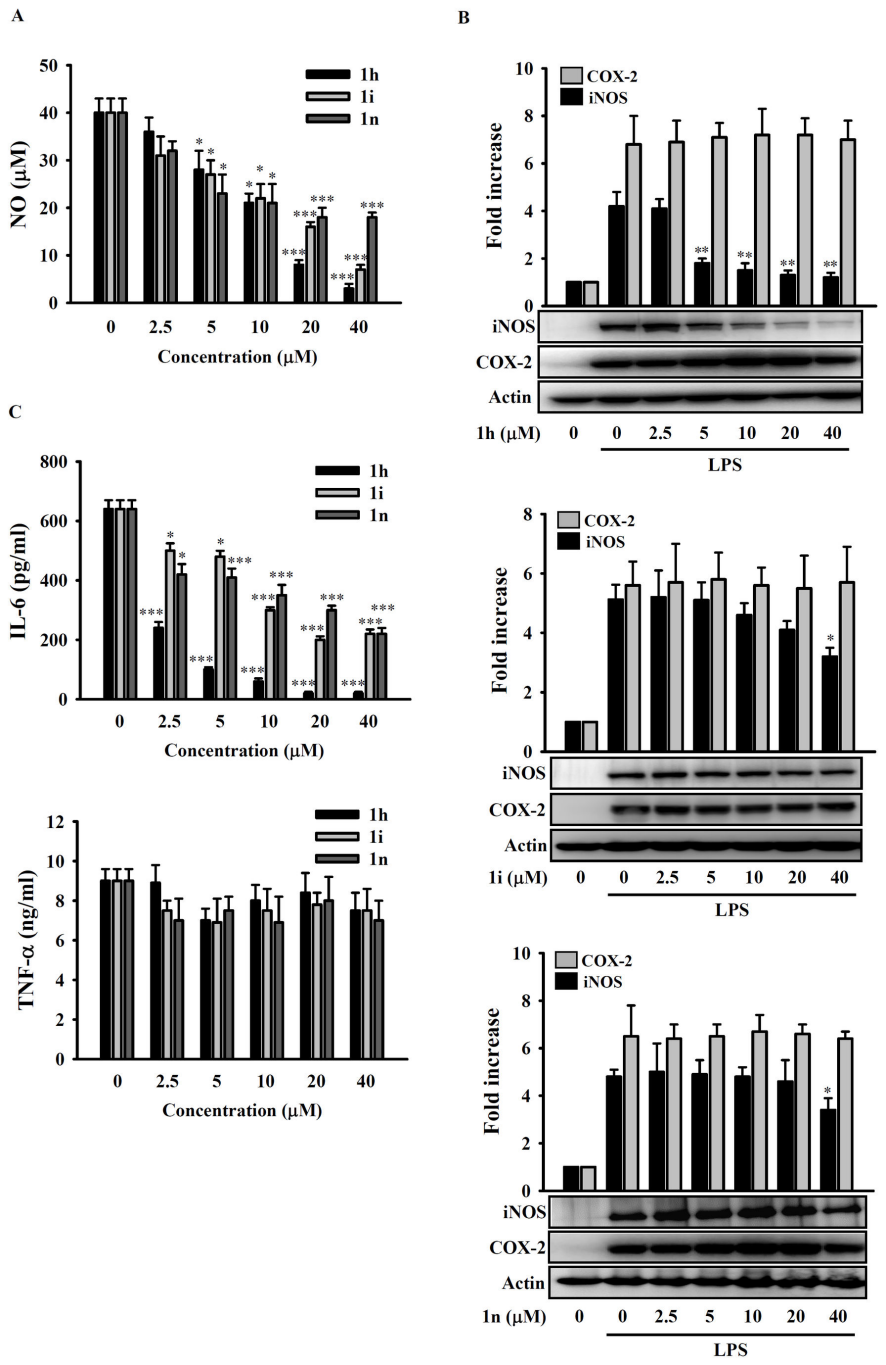
Effect of polyenylpyrrole derivatives on the expression of inflammatory mediators in LPS-stimulated RAW 264.7 macrophages. In (**A**) and (**C**), the cells (5 × 10^5^/ml; 1 ml) were incubated for 30 min with 2.5-40 µM compound **1h**, **1i**, or **1n** or DMSO (vehicle), then LPS (1 µg/ml) was added and incubation continued for 24 h, then NO (**A**) or IL-6 or TNF-α (**C**) in the culture medium was assayed by the Griess reaction or ELISA, respectively. In (**B**), cells (5 × 10^5^/ml; 1 ml) were pretreated for 30 min with 2.5-40 µM compound **1h** or DMSO, then LPS (1 µg/ml) was added and incubation continued for 24 h, then expression of iNOS and COX-2 was measured by Western blotting. The fold increase is the intensity of the band of interest divided by that of the actin band normalized to the corresponding value for the 0 LPS/0 inhibitor control. In (**A**) and (**C**), the data are expressed as the mean ± SD for three separate experiments, while, in (**B**), the results are representative of those obtained in three different experiments and the histogram shows the quantification expressed as the mean ± SD for these 3 experiments. *, **, and *** indicate a significant difference at the level of *p* < 0.05, *p* < 0.01, or *p* < 0.001, respectively, compared to the DMSO/LPS group.

To investigate the inhibitory effect of compounds **1h, 1i**, and **1n** on the LPS-induced inflammatory response, NO levels in the supernatants of RAW 264.7 macrophages incubated with DMSO (vehicle) or compound **1h, 1i**, or **1n** for 30 min before, and during, incubation for 24 h with LPS (1 µg/ml) were measured by the Griess reaction. The results showed that treatment with compound **1h, 1i**, or **1n** alone did not alter NO levels produced by non-activated cells (data not shown), but decreased NO production by LPS-activated cells in a dose-dependent manner (Figure **2A**). We next investigated their effect on the expression of iNOS, the NO producing enzyme, using Western blots. As shown in Figure **2B**, pretreatment of RAW 264.7 macrophages for 30 min with 0-40 µM compound **1h** (**top panel**), **1i** (**center panel**), or **1n** (**bottom panel**) before addition of LPS for 24 h resulted in reduced LPS-induced iNOS expression, the effect being significant at 5-40 µM compound **1h** and 40 µM compound **1i** or **1n**, but did not affect expression of COX-2, an enzyme producing prostaglandin E2. In addition, we tested the effect of pretreatment on cytokine secretion (IL-6 and TNF-α) by LPS-activated RAW 264.7 macrophages and found that none of the three compounds altered background levels of IL-6 and TNF-α in non-stimulated macrophages (data not shown), but all three significantly decreased IL-6 production by LPS-activated cells in a dose-dependent manner, with compound **1h** being more potent than **1i** and **1n** (Figure **2C** **, upper panel**), while TNF-α secretion was only slightly and non-significantly reduced (Figure **2C** **, lower panel**).

### Compound 1h decreases IL-6 and TNF-α secretion by LPS-activated J774A.1 macrophages, peritoneal macrophages, and JAWSII dendritic cells

**Figure 3:**
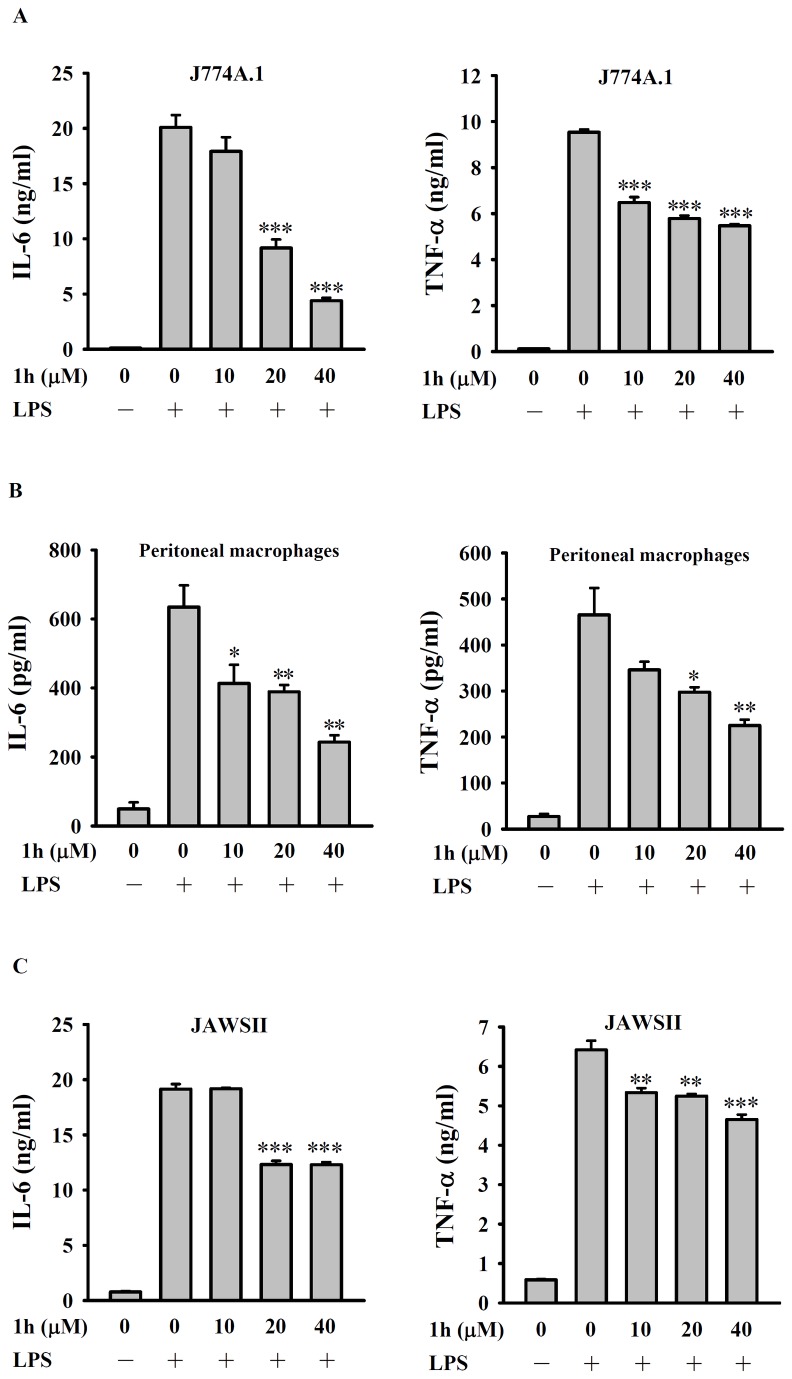
Effect of compound 1h on LPS-induced secretion of IL-6 and TNF-α by J774A.1 macrophages, peritoneal macrophages, and JAWSII dendritic cells. (**A**) J774A.1 macrophages, (**B**) peritoneal macrophages, or (**C**) JAWSII dendritic cells (all 4 × 10^5^/ml; 1 ml) were incubated for 30 min with 10-40 µM compound **1h** or DMSO, then LPS (1 µg/ml) was added and incubation continued for 24 h, then IL-6 levels (**left panels**) and TNF-α levels (**right panels**) in the culture medium were measured by ELISA. The data are expressed as the mean ± SD for three separate experiments. *, **, and *** indicate a significant difference at the level of *p* < 0.05, *p* < 0.01, or *p* < 0.001, respectively, compared to the DMSO/LPS group.

To confirm the anti-inflammatory activity of compound **1h** seen with RAW 264.7 cells, its effect on LPS-induced cytokine secretion was investigated using another murine macrophage cell line J774A.1 and primary peritoneal macrophages from C57BL/6 mice and the results showed that it reduced secretion of IL-6 and TNF-α in both J774A.1 cells (Figure **3A**) and peritoneal macrophages (Figure **3B**) in a dose-dependent manner. It also reduced LPS-induced IL-6 and TNF-α secretion by the murine dendritic cell line JAWSII (Figure **3C**).

### Compound 1h reduces IL-1β secretion by inhibiting the NLRP3 inflammasome

**Figure 4:**
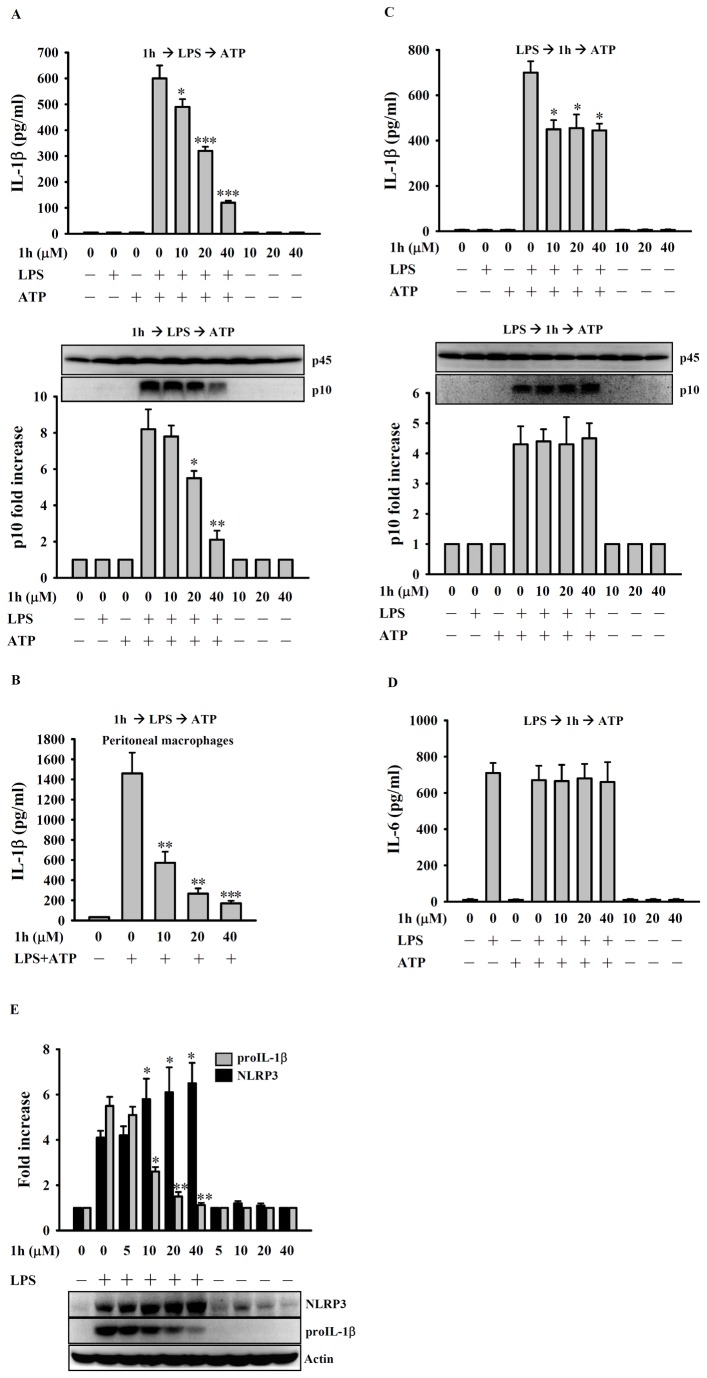
Effect of compound 1h on NLRP3 inflammasome activation in LPS+ATP-activated J774A.1 macrophages. (**A**) J774A.1 macrophages (1 × 10^6^/ml; 1 ml) or (**B**) peritoneal macrophages (1 × 10^5^/ml; 1 ml) were incubated with 10-40 µM compound **1h** or DMSO for 30 min, then LPS (1 µg/ml) was added and incubation continued for 5.5 h, then the cells were stimulated with ATP (5 mM) for an additional 30 min, then IL-1β in the culture medium was measured by ELISA (**A, upper panel; B**) and levels of active caspase-1 (p10) (**A, lower panel**) measured by Western blotting. In (**C**) and (**D**), J774A.1 macrophages (1 × 10^6^/ml; 1ml) were incubated with LPS (1 µg/ml) for 5.5 h, then with 10-40 µM compound **1h** or DMSO for 30 min in the continued presence of LPS, followed by stimulation with ATP (5 mM) for an additional 30 min, then IL-1β levels (**C, upper panel**) and IL-6 levels (**D**) in the culture medium were measured by ELISA and levels of active caspase-1 (p10) were measured by Western blotting (**C, lower panel**). In A and C, the fold increase is the intensity of the p10 band divided by that of the p45 band normalized to the corresponding value for the 0 LPS/0 inhibitor control. In (**E**), J774A.1 macrophages (1 × 10^6^/ml; 1 ml) were incubated for 30 min with DMSO or 1-40 µM compound **1h**, then LPS (1 µg/ml) was added and incubation continued for 6 h, then expression of NLRP3 and proIL-1β was measured by Western blotting. The fold increase is the intensity of the band of interest divided by that of the actin band normalized to the corresponding value for the 0 LPS/0 inhibitor control. In the ELISA studies, the data are expressed as the mean ± SD for three separate experiments, while, in the Western blot studies, the results shown are representative of those obtained in three different experiments and the histogram shows the quantification expressed as the mean ± SD. *, **, and *** indicate a significant difference at the level of *p* < 0.05, *p* < 0.01, and *p* < 0.001, respectively, compared to the DMSO/LPS/ATP group (**A**, **B**), LPS/DMSO/ATP group (**C**, **D**), or the DMSO/LPS group (**E**).

ATP is known to activate the NLRP3 inflammasome in LPS-primed macrophages, leading to caspase-1 activation and IL-1β secretion [28]. To examine whether compound **1h** could affect NLRP3 inflammasome activation, the mouse macrophage cell line J774A.1 was used (RAW 264.7 macrophages are not suitable for studying the NLRP3 inflammasome). The full activation of the NLRP3 inflammasome requires both a priming signal (LPS) and an activation signal (ATP), and we therefore examined the effect of compound **1h** on both signaling events. As shown in Figure **4A**, incubation of J774A.1 macrophages with compound **1h** for 30 min before treatment for 5.5 h with LPS, followed by treatment with ATP for 30 min significantly inhibited IL-1β secretion (**upper panel**) and the generation of active caspase-1 (p10; **lower panel**) in a dose-dependent manner. Using the same conditions, compound **1h** also inhibited IL-1β secretion by primary peritoneal macrophages (Figure **4B**). In addition, to examine whether compound **1h** was able to affect the ATP-mediated activation signal, we incubated J774A.1 cells with LPS for 5.5 h, then with compound **1h** for 30 min before ATP stimulation and, as shown in Figure **4C**, found that compound **1h** inhibited the LPS-induced increase in IL-1β secretion (**upper panel**), but not caspase-1 activation (**lower panel**), but, as shown in Figure **4D**, had no significant effect on IL-6 secretion. These results demonstrate that compound **1h** inhibited NLRP3 inflammasome-mediated IL-1β secretion, but not IL-6 secretion, which is independent of the NLRP3 inflammasome. We also tested the ability of compound **1h** to inhibit expression of NLRP3 protein (an essential component of the NLRP3 inflammasome) and of proIL-1β (IL-1β precursor) in LPS-activated J774A.1 cells by incubating the cells with different concentrations of compound **1h** for 30 min before addition of LPS for another 6 h and, as shown in Figure **4E**, found that it inhibited LPS-induced proIL-1β expression in a dose-dependent fashion, but increased NLRP3 expression.

### Compound 1h inhibits ROS production by, and MAPK activation in, LPS-activated macrophages

**Figure 5:**
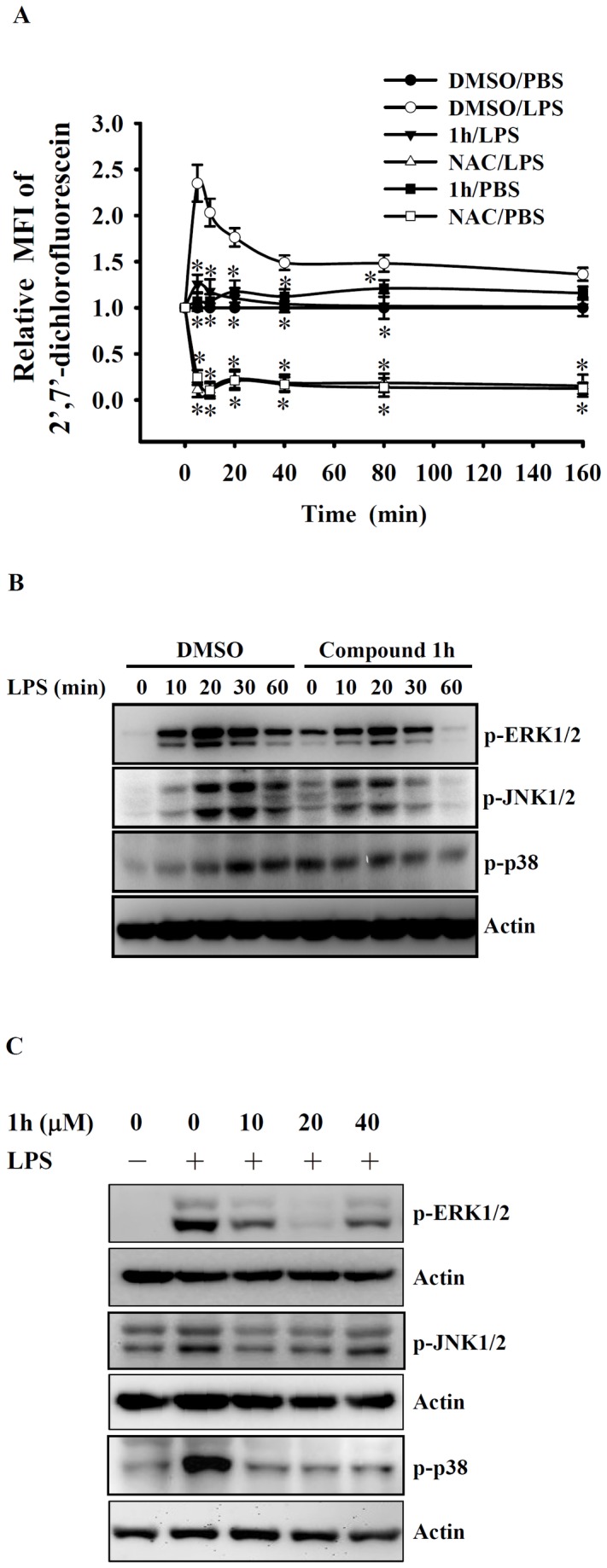
Effect of compound 1h on ROS production and MAPK phosphorylation in LPS-activated macrophages. In (**A**), RAW 264.7 macrophages (5 × 10^5^/ml; 1 ml) were incubated for 30 min with compound **1h** (20 µM), N-acetyl cysteine (NAC; 10 mM), or DMSO (vehicle), then 2’, 7’-dichlorofluorescein diacetate (2 µM) was added for 30 min, followed by LPS (1 µg/ml) stimulation for the indicated time, then ROS levels were measured by detection of the mean fluorescence intensity (MFI) of the fluorophore carboxyl-DCF and expressing this value relative to that at time zero. In (**B**), RAW 264.7 macrophages (5 × 10^5^/ml; 1 ml) were incubated for 30 min with compound **1h** (20 µM) or DMSO, then LPS (1 µg/ml) was added and incubation continued for 0-60 min, then phosphorylation of ERK1/2, JNK1/2, and p38 was analyzed by Western blotting and expressed relative to actin expression and as a fold increase compared to the control group at 0 time. In (**C**), J774A.1 macrophages (5 × 10^5^/ml; 1 ml) were incubated for 30 min with 10-40 µM compound **1h** or DMSO, then LPS (1 µg/ml) was added and incubation continued for 20 min, then phosphorylation of ERK1/2, JNK1/2, and p38 was analyzed as in B. In (**A**), the data are expressed as the mean ± SD for three separate experiments, while, in (**B**) and (**C**), the results are representative of those obtained in three different experiments. * indicates a significant difference at the level of *p* < 0.05 compared to the DMSO/LPS group.

ROS have been demonstrated to play important roles in LPS-mediated cytokine expression [14,26]. To test whether compound **1h** exerted its anti-inflammatory effect on LPS-activated cells by downregulation of ROS production, intracellular ROS production in LPS-activated RAW 264.7 macrophages was measured. As shown in the time-course study in Figure **5A** LPS stimulation of cells rapidly induced ROS production and pretreatment for 30 min with NAC (10 mM), a potent antioxidant, reduced ROS production. Pretreatment for 30 min with compound **1h** (20 µM) also reduced LPS-stimulated ROS production, suggesting that its anti-inflammatory effect might be mediated partially through its antioxidative activity.

LPS is a potent inducer of macrophage activation and pro-inflammatory cytokine production, as it activated TLR4, which, in turn, activates many signaling pathways, including the mitogen-activated protein kinase (MAPK) signaling pathways [29]. To examine whether the effects of compound **1h** on LPS-induced macrophages were associated with activation of MAPK signaling cascades, RAW 264.7 macrophages were incubated with DMSO or compound **1h** (20 µM) for 30 min, then with LPS (1 µg/ml) for 0-60 min, and phosphorylation of the MAPKs, ERK1/2, JNK1/2, and p38 determined by Western blot analysis. As shown in Figure **5B**, compound **1h** inhibited phosphorylation of all 3 MAPKs in LPS-activated RAW 264.7 macrophages, these effects being maximal with 20-30 min of LPS stimulation. Using LPS stimulation for 20 min, these results were confirmed in J774A.1 macrophages (Figure **5C**). These results show that compound **1h** inhibits activation of the MAPK signaling cascades in LPS-activated macrophages.

### Compound 1h inhibits NF-κB activation in LPS-activated macrophages

**Figure 6:**
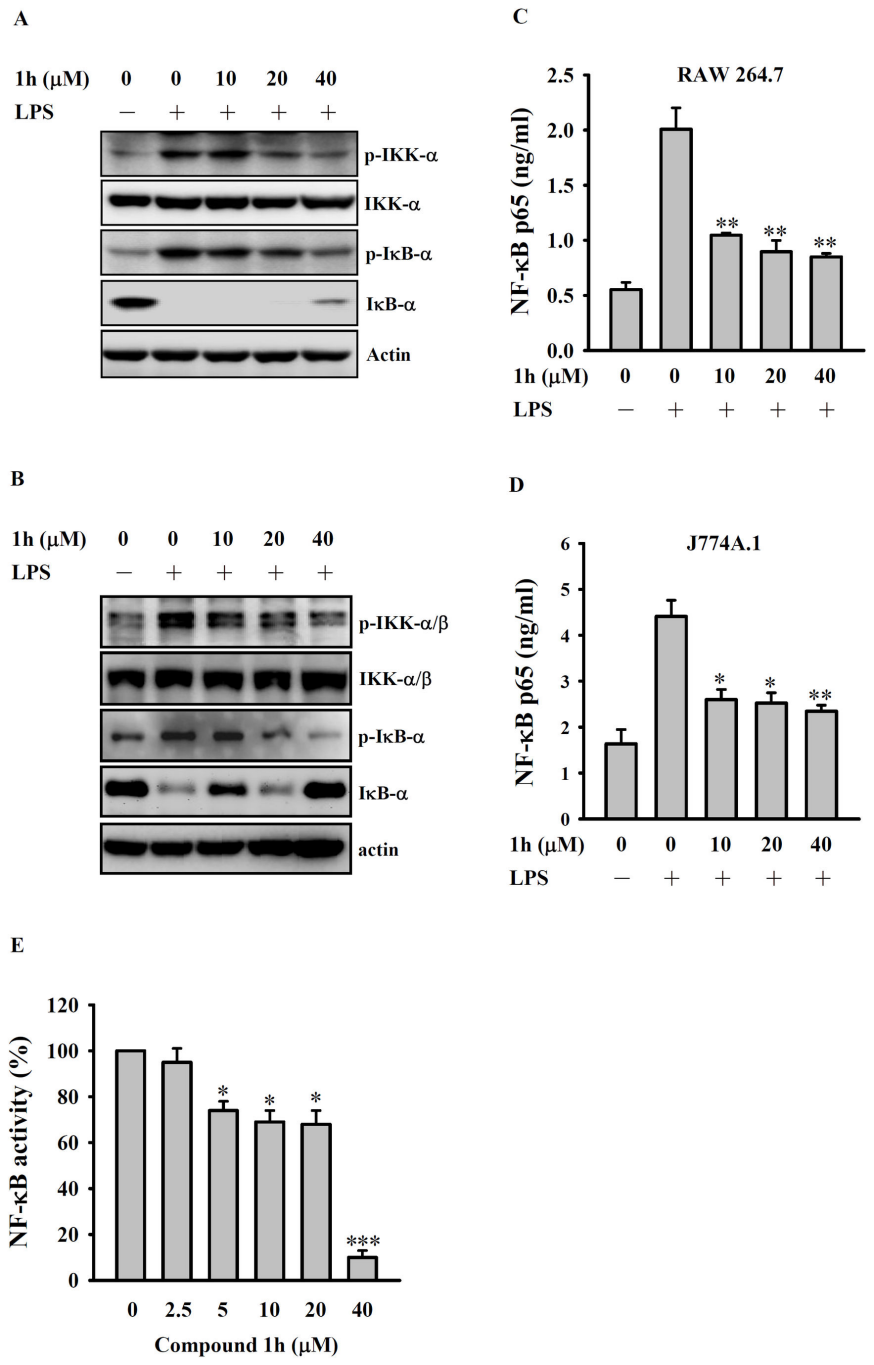
Effect of compound 1h on NF-κB activation in LPS-activated macrophages. (**A**) RAW 264.7 macrophages or (**B**) J774A.1 macrophages (both 5 × 10^5^/ml; 1 ml) were incubated for 30 min with 10-40 µM compound **1h** or DMSO, then LPS (1 µg/ml) was added and incubation continued for 20 min, then levels of phosphorylated and total IKK-α and IκB-α were measured by Western blotting. (**C**) RAW 264.7 macrophages or (**D**) J774A.1 macrophages (both 5 × 10^5^/ml; 1 ml) were treated as in A and B, then nuclear translocation of NF-κB was analyzed by ELISA. (**E**) RAW-Blue^TM^ cells (5 × 10^5^/ml; 1 ml) were incubated for 30 min with 2.5-40 µM compound **1h** or DMSO, then LPS (1 µg/ml) was added and incubation continued for 24 h, then SEAP activity was measured by the QUANTI-Blue^TM^ assay and expressed as a percentage of that in the absence of compound **1h**. In (**A**) and (**B**), the results are representative of those obtained in three different experiments. In (**C**-**E**), the data are expressed as the mean ± SD for three separate experiments. *, **, and *** indicate a significant difference at the level of *p* < 0.05, *p* < 0.01, and *p* < 0.001, respectively, compared to the DMSO/LPS group.

In resting macrophages, NF-κB is sequestered in the cytoplasm as an inactive precursor complex by its inhibitory protein, IκB. Following LPS stimulation, IκB in the complex is phosphorylated by IκB kinase (IKK), ubiquitinated, and rapidly degraded in proteasomes, thus releasing NF-κB [30]. In determining whether compound **1h** could inhibit LPS-stimulated NF-κB signaling in macrophages, we found that it inhibited phosphorylation of IKK-α and IκB-α in a dose-dependent manner and also had a inhibitory effect on IκB-α degradation in LPS-activated RAW 264.7 macrophages (Figure **6A**) and J774A.1 macrophages (Figure **6B**). In addition, it inhibited NF-κB nuclear translocation in LPS-activated RAW 264.7 macrophages (Figure **6C**) and J774A.1 macrophages (Figure **6D**). Furthermore, using NF-κB-dependent alkaline phosphatase reporter cells, we demonstrated that NF-κB transcriptional activity in LPS-stimulated macrophages was also reduced by compound **1h** (Figure **6E**). These results show that compound **1h** inhibits the activation of the NF-κB signaling cascades in LPS-activated macrophages.

### Compound 1h inhibits ROS production by, and PKC-α phosphorylation in, ATP-activated macrophages

**Figure 7:**
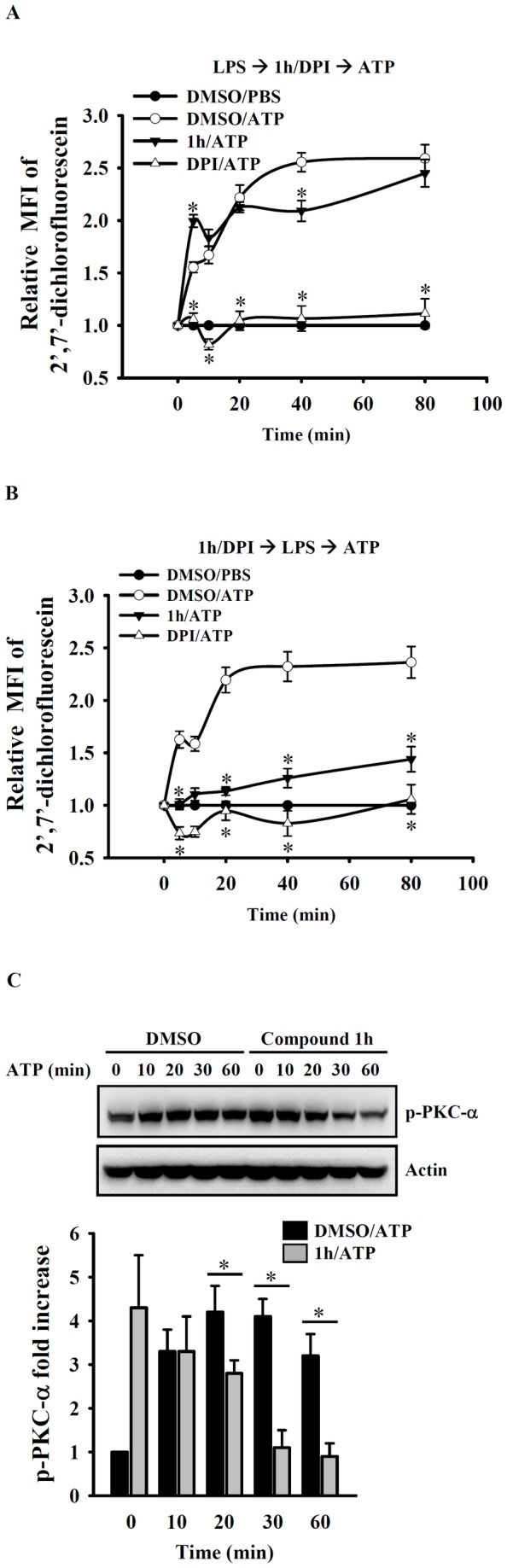
Effect of compound 1h on ROS production and PKC-α phosphorylation in ATP-activated macrophages. In (**A**), J774A.1 macrophages (1 × 10^6^/ml; 1 ml) were incubated with LPS (1 µg/ml) for 6 h, then with compound **1h** (20 µM), the NADPH oxidase inhibitor DPI (25 µM), or DMSO (vehicle) for 30 min in the continued presence of LPS, then 2’,7’-dichlorofluorescein diacetate (2 µM) was added for 30 min, followed by ATP (5 mM) for the indicated time, then ROS levels were determined by measuring the mean fluorescence intensity (MFI) of the fluorophore carboxyl-DCF and expressing this value relative to that at time zero. In (**B**), J774A.1 macrophages (1 × 10^6^/ml; 1 ml) were incubated with compound **1h** (20 µM), DPI (25 µM), or DMSO (vehicle) for 30 min, then LPS (1 µg/ml) was added for 6 h; the cells were then incubated with 2’,7’-dichlorofluorescein diacetate (2 µM) for 30 min, then with ATP (5 mM) for the indicated time and ROS levels were measured by detection of the fluorescence intensity of the fluorophore carboxyl-DCF and expressed relative to that at time zero. In (**C**), LPS-primed J774A.1 macrophages (1 × 10^6^/ml; 1 ml) were incubated for 30 min with 20 µM compound **1h** or DMSO (vehicle) followed by ATP (5 mM) stimulation for 0-60 min, then phosphorylation of PKC-α was analyzed by Western blotting and expressed as the fold increase measured as the intensity of the PKC-α band divided by that of the actin band normalized to the corresponding value for DMSO at 0 minutes. In (**A**) and (**B**), the data are expressed as the mean ± SD for three separate experiments, while, in (**C**), the results are representative of those obtained in three different experiments and the histogram shows the quantification expressed as the mean ± SD. * indicates a significant difference at the level of *p* < 0.05 compared to the DMSO/ATP group.

ATP-induced ROS production by NADPH oxidase is required for caspase-1 activation in, and IL-1β secretion by, macrophages [31,32]. To determine whether the inhibition of LPS-induced IL-1β secretion by compound **1h** occurred via inhibition of ATP-induced ROS production, LPS-primed J774A.1 macrophages were incubated with vehicle or compound **1h** (20 µM) for 30 min before addition of ATP or PBS for 0-40 min and the results showed that compound **1h** slightly reduced ATP-induced ROS production at 40 min (Figure **7A**), whereas addition of compound 1h 30 min before LPS priming significantly inhibited ATP-induced ROS production (Figure **7B**), while the NADPH oxidase inhibitor, diphenylene iodonium (DPI) inhibited both processes. In addition, when LPS-primed J774A.1 macrophages were incubated with vehicle or compound **1h** (20 µM) for 30 min before ATP stimulation for 0-60 min, compound **1h** caused significant inhibition of ATP-induced PKC-α phosphorylation at 20-60 min (Figure **7C**).

## Discussion

Fungi are a valuable source of novel natural products with many biological activities [8,33]. Polyketides isolated from fungi exhibit various biological properties, such as antibacterial [5], antifungal [5,6], and antitumor [7,8] activities, but their effect on immune responses are not fully understood. It has been demonstrated that mycolactone, a natural polyketide produced by *Mycobacterium ulcerans* which causes the skin disease Buruli ulcer, reduces the immune response and the infiltration of inflammatory cells into the infection site [10]. In addition, a polyketide synthase-produced phenolic glycolipid isolated from *Mycobacterium tuberculosis* has been found to inhibit the release of pro-inflammatory mediators by activated macrophages [11]. Commercially available polyketides include rapamycin, a potent immunosuppressant [34], and fumagillin, which has been used in the treatment of microsporidiosis [35]. These results indicate that polyketides could be a valuable source of anti-inflammatory agents.

Thus far, polyketides have been mainly isolated from fungi or bacteria, but the typically small quantities that can be obtained in this way often limit biological studies. To address this limitation, we previously synthesized a group of polyketides (Figure **1** and Table **1**) consisting of two hit compounds isolated from the soil ascomycete *Gymnoascus reessii*, auxarconjugatin A (compound **1b**) and *12E*-isorumbrin (compound **1e**), and several analogs and evaluated their anti-lung cancer activity [9]. In the present study, we evaluated the anti-inflammatory activities of these compounds. Although compounds **1a-g** were cytotoxic, compounds **1h-n** were able to inhibit LPS-induced NO production without reducing macrophage viability.

ROS have an established role in inflammatory cytokine production in response to LPS [14,36]. They have also been implicated as playing an important role in NLRP3 inflammasome activation [25,37-39]. Compound **1h** exhibited antioxidative activity by reducing LPS-induced ROS production. However, from the data obtained, we were unable to conclude whether it inhibits the enzymes involved in ROS production or scavenges the ROS produced. However, further studies indicated that its effect of decreasing LPS-induced production of iNOS, NO, and IL-6 may be, at least in part, due to its antioxidative activity. The effect of compound **1h** on LPS-induced TNF-α secretion might be cell type-dependent, as it reduced TNF-α secretion by LPS-activated murine J774A.1 macrophages, primary mice peritoneal macrophages, and JAWSII murine dendritic cells, but not LPS-activated murine RAW 264.7 macrophages. TNF-α secretion is controlled at the transcriptional and post-transcriptional levels by NF-κB and TNF-α converting enzyme, respectively (29,30). Since compound **1h** reduced LPS-induced NF-κB activation in both J774A.1 and RAW 264.7 macrophages, this suggests that NF-κB plays less of a role in TNF-α secretion by RAW 264.7 macrophages. An earlier study reported that NLRP3 mRNA expression in LPS-activated macrophages was inhibited by ROS inhibitors [40], supporting an important role of ROS in NLRP3 expression. However, our results seem to contradict this finding, as, although compound **1h** reduced ROS production in LPS-activated cells, it did not reduce NLRP3 protein expression. We therefore speculate that LPS activates signaling pathways other than the ROS pathway for the regulation of NLRP3 protein expression. We also found that compound **1h** was able to reduce not only conventional inflammatory responses, such as NO and IL-6 production, but also NLRP3 inflammasome-mediated IL-1β expression in LPS-activated macrophages. NLRP3 inflammasome activation required both a priming signal (e.g., from TLR4) and an activation signal (e.g., from ATP) for caspase-1 activation and IL-1β secretion [40,41]. In the LPS-mediated priming stage, although compound **1h** was not able to inhibit NLRP3 expression in LPS-activated macrophages, it significantly inhibited both proIL-1β expression and ROS production. These results show that it inhibits NLRP3 inflammasome activation by reducing ROS production, but not by reducing NLRP3 protein expression. Addition of compound **1h** after LPS priming only slightly reduced ATP-induced ROS production (Figure **7A**), but addition before LPS priming significantly reduced LPS+ATP-induced ROS productionOK (Figure **7B**). These results suggest that it blocks an as yet unknown signal induced by LPS that contributes to ATP-mediated ROS production. They also explain why compound **1h** significantly inhibited caspase-1 activation and IL-1β secretion when added before LPS priming, but only slightly reduced IL-1β secretion when added after LPS priming. ATP-induced ROS production has been shown to activate caspase-1 through the PI3-kinase/AKT pathway [32]. However in our study, we found that compound **1h** did not reduce ATP-induced AKT phosphorylation in LPS-primed macrophages (data not shown), but, instead resulted, in a reduction in ATP-induced PKC-α phosphorylation.

**Figure 8:**
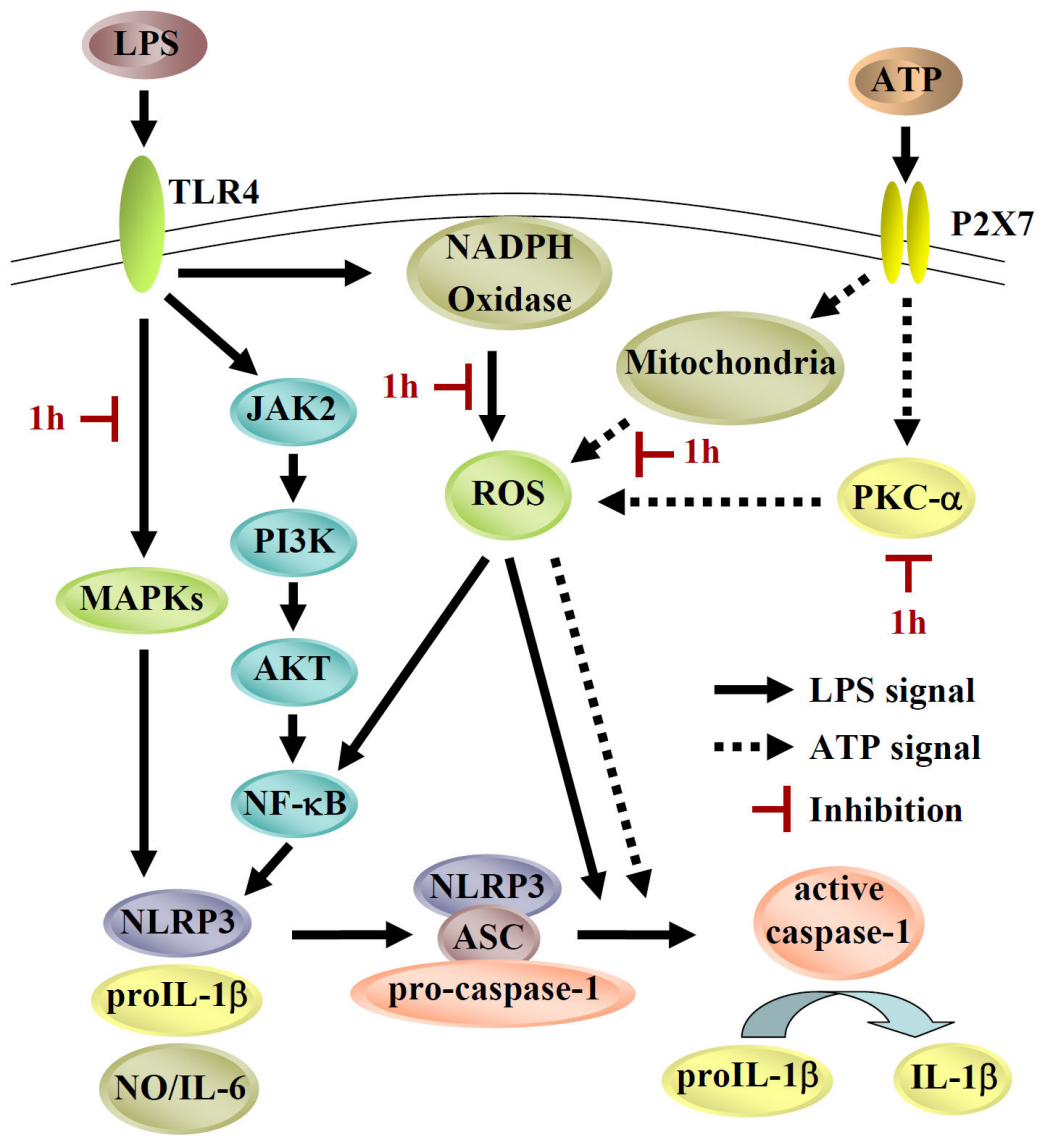
Proposed anti-inflammatory mechanism of compound 1h in LPS+ATP-activated macrophages.

In summary, we have shown that compound **1h**, a non-toxic polyenylpyrrole, is able to inhibit NLRP3 inflammasome activation and NO and IL-6 expression by inhibiting LPS- and ATP-induced ROS production and LPS-induced activation of MAPK and NF-κB. The proposed anti-inflammatory mechanism of compound **1h** is shown in Figure **8**. These results suggest that compound **1h** could be a lead compound for the development of anti-inflammatory therapeutics.
